# Spatio-Temporal Progression of Grey and White Matter Damage Following Contusion Injury in Rat Spinal Cord

**DOI:** 10.1371/journal.pone.0012021

**Published:** 2010-08-09

**Authors:** C. Joakim Ek, Mark D. Habgood, Jennifer K. Callaway, Ross Dennis, Katarzyna M. Dziegielewska, Pia A. Johansson, Ann Potter, Benjamin Wheaton, Norman R. Saunders

**Affiliations:** Department of Pharmacology, University of Melbourne, Parkville, Victoria, Australia; University of North Dakota, United States of America

## Abstract

Cellular mechanisms of secondary damage progression following spinal cord injury remain unclear. We have studied the extent of tissue damage from 15 min to 10 weeks after injury using morphological and biochemical estimates of lesion volume and surviving grey and white matter. This has been achieved by semi-quantitative immunocytochemical methods for a range of cellular markers, quantitative counts of white matter axonal profiles in semi-thin sections and semi-quantitative Western blot analysis, together with behavioural tests (BBB scores, ledged beam, random rung horizontal ladder and DigiGait™ analysis). We have developed a new computer-controlled electronic impactor based on a linear motor that allows specification of the precise nature, extent and timing of the impact. Initial (15 min) lesion volumes showed very low variance (1.92±0.23 mm^3^, mean±SD, n = 5). Although substantial tissue clearance continued for weeks after injury, loss of grey matter was rapid and complete by 24 hours, whereas loss of white matter extended up to one week. No change was found between one and 10 weeks after injury for almost all morphological and biochemical estimates of lesion size or behavioural methods. These results suggest that previously reported apparent ongoing injury progression is likely to be due, to a large extent, to clearance of tissue damaged by the primary impact rather than continuing cell death. The low variance of the impactor and the comprehensive assessment methods described in this paper provide an improved basis on which the effects of potential treatment regimes for spinal cord injury can be assessed.

## Introduction

Spinal cord trauma is often devastating for the patients as it can cause permanent loss of motor, sensory and autonomic nervous system functions below the level of the injury. However, not all of the damage to the spinal cord occurs at the time of the injury. Typically, there is an initial destruction of tissue (primary injury) at the time of impact followed by a period of further damage due to structural, cellular, biochemical and immunological changes in the region surrounding the primary injury, processes that are usually referred to as “secondary” injury. A substantial amount of work has been published on secondary injury following trauma to brain [1], [2], [3] and spinal cord [1], [4]. There are currently no effective treatments available to reverse spinal cord damage and restore lost function despite concentrated efforts over several decades [1], [5], [6]. Thus limiting ongoing secondary damage has the best immediate prospect for improving patient outcomes following traumatic spinal injury. Secondary injury processes following trauma are generally considered to last for many days, or even weeks (as reviewed in [1]) and many treatments are aimed at ameliorating these progressive effects [4]. However, from an examination of the literature it is apparent that pathological processes following spinal cord injury, which are involved in clearing damaged tissue resulting from the primary injury and progressive cyst formation, may not be clearly distinguished from further loss of grey and/or white matter due to apoptosis and continuing axonal degeneration in the subsequent days and weeks. Injury-induced disruption of the vascular supply and the ensuing hypoxia and ischaemia is widely regarded as the central initiator of the cascade of events underlying secondary tissue damage [4], [7]–[11]. Understanding which tissue is at risk of secondary destruction following spinal cord injury and the time course over which this damage occurs, is critical for the design and assessment of therapies aimed at limiting consequences of trauma. To study the progression of spinal cord damage over extended periods of time when only terminal measurements can be made, requires an animal model that produces consistent sized injuries. In this paper we describe a contusion model with low variance, the progression in lesion size, neuron numbers and myelinated axon numbers, as well as various biochemical parameters and behavioural performance, in the hours, days and weeks following a spinal injury in young adult rats. The results show that following spinal cord contusion injury of the type induced in this study, the size of the primary injury shortly after it was made was of very low variance. The subsequent loss of grey matter in and around the lesion site did not continue beyond 24 hours after injury, whereas there was a loss of white matter spreading out from the centre of the lesion site that continued for up to one week after injury. Consistent with these observations, no change was found between one and 10 weeks after injury for most of the morphological and biochemical estimates of lesion size or quantitative behavioural methods used.

## Methods

### Ethics statement

All animal experiments were conducted following NH&MRC guidelines and were approved by the University of Melbourne Animal Ethics Committee (Ethics#0703702).

### Impactor device

**Figure 1:**
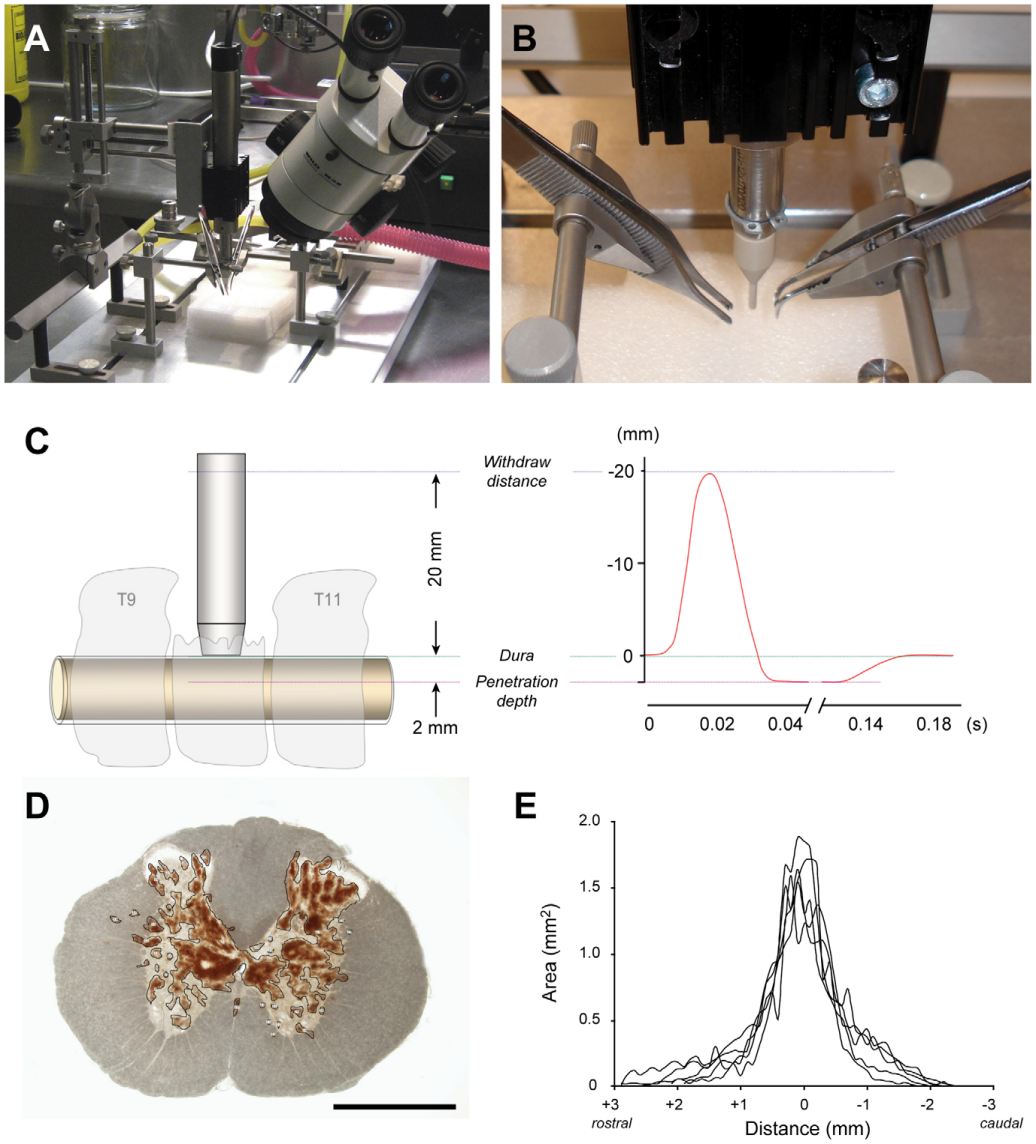
Spinal impactor device and measurements of initial injury volume. **A&B**) The linear motor of the impactor is mounted on a stereotaxic frame and a servo-controller unit used to deliver a precisely controlled impact to the exposed spinal cord of adult rats. The vertebral column is stabilised by clamps attached to the dorsal spines of the vertebrae either side of the laminectomy site to prevent movement of the vertebral column on impact. **C**) Schematic diagram of the impactor device in operation. The tip is lowered manually using the manipulator arm controls until it is just touching the surface of the dura mater exposed via a laminectomy (left). The tip withdraw height, acceleration rate, impact velocity, depth of tip penetration, deceleration rate and dwell time are preset by the user in the control software. The position of the tip during the course of each impact is recorded in real-time and graphically displayed to confirm the impact parameters (right). **D**) Vibrating microtome section (100 µm) through the centre of the lesion site in a spinal cord from an animal at 15 min after injury. The section has not been stained and was viewed under transmitted light. The areas of visibly damaged tissue and haemorrhage (brown colour) in each section were outlined and measured. Note the extensive damage and bleeding in the central grey matter of the cord and undamaged appearance of the surrounding white matter. The impactor tip strikes vertically down onto the dorsal columns (arrow) and penetrates to a preset depth of 2.0 mm below the surface of the dura mater. Scale bar is 1 mm. **E**) Lesion area measurements in serial sections (100 µm thick) spanning the entire injury site from animals subjected to a single impact injury at T10. The spinal cords were collected 15 min after injury and each line represents a single animal. The mean lesion volume was 1.92±0.23 mm^3^ (SD, n = 5) with a range of 1.52 to 2.10 mm^3^.

The impactor device used to produce lower thoracic spinal injuries (Fig. 1A, B) was developed from the device described previously by Bilgen [12] and Narayana et al. [13]. It comprises a LinMot linear motor (model number PS01-23×80) and slider (model number PL01-12×170/120) mounted on a manipulator of a stereotaxic frame, a LinMot servo controller unit (model number E1100-RS) and a PC computer running LinMot control software (LinTalk Version 3.6). Small autoclavable tapered plastic tip pieces were fabricated and fitted to the end of the slider to provide a flat impacting surface (2 mm diameter). The key feature of this device is that it allows the user to control the depth of penetration of the impact. The linear motor uses electromagnetic force to propel a metal rod (slider) along the central hollow tube of the motor casing. The computer controlled servo unit allows the user to specify the precise nature and timing of the movements made by the slider (positions to move to, slider velocity, acceleration rate, deceleration rate, times to remain stationary). Rapid and complex movement patterns can be pre-programmed into the motor and automatically executed upon receipt of a trigger signal from the controller. In operation, the motor is first initialised and the slider extended to a pre-defined starting position relative to the motor casing (30 mm extended). The manipulator on which the motor is mounted is then used to manually position the tip of the impactor so that it is just touching the surface of the tissue that is to be impacted (Fig. 1C). Once in position, the computer software is used to operate the impactor and deliver the impact. The position of the slider is monitored in real-time and displayed graphically allowing the actual impact velocity and tip penetration depth to be recorded (Fig. 1C). The impactor device can be fitted to most stereotaxic frames and can be used for both spinal cord injury or head/brain injury studies (see Fig. 1A, B). It can also be used for larger animals by using larger linear motors and a larger probe tip.

### Surgery and spinal cord injury

Young adult male Sprague-Dawley rats (body weight range 170–200 g) were anaesthetised with 3% isoflurane in oxygen delivered via a facemask fitted over the mouth and nose of the animals. Rectal temperature was monitored and maintained constant (37.5±0.2°C SD) by placing the rats on a temperature regulated pad. The fur over the lower thoracic and lumbar vertebral column was shaved and the area cleaned with chlorhexidine in 70% alcohol. A longitudinal incision was made through the lower thoracic skin to expose the underlying musculature and the T9–T11 vertebrae identified. The muscles inserted on the vertebral spines of the T9–T11 vertebrae were detached and a laminectomy performed to remove the dorsal aspect of the T10 vertebrae. The animal was transferred to a spinal stereotaxic frame and clamps attached to the T9 and T11 vertebral spines to secure the vertebral column. The tip of the impactor was positioned above the T10 laminectomy site and manually lowered using the manipulator controls until the tip of the impactor was just touching the surface of the exposed dura mater (viewed under a stereomicroscope). A single impact trauma was delivered using the computer controller. The depth of penetration (2 mm) of the impactor tip from its starting position, the target velocity of the impactor (5 m/s) and dwell time (100 ms) were preset in the control software (Fig. 1). After delivery of the impact, the injury site was inspected using a stereomicroscope to confirm that the cord had been injured. In most cases, visible bruising of the spinal cord could be seen, but in a few animals bleeding into the sub-dural space above the spinal cord obscured direct visibility of the cord. The sham operated control animals received the same surgery and were mounted in the stereotaxic frame, but no impact was delivered. The laminectomy site was closed with three layers of sutures through the adjacent vertebral musculature, the subdermal tissues and finally through the overlying skin. The area was cleaned again with chlorhexidine in 70% alcohol. Sterile saline (5 mL/kg) and analgesic (buprenorphine 0.06 mg/kg) were administered by intraperitoneal injection and the animal placed into a rat cage on a heated pad to recover from the anaesthesia. Once fully conscious, the animals were examined to confirm paralysis of the hindlimbs. Additional injections of sterile saline and analgesic were administered once daily for the first 4 days after injury. Animals were excluded from the study only if there was no visible damage to the spinal cord and they were able to use their hindlimbs immediately on recovery from anaesthesia. A total of 3 out of 68 injured animals were excluded. The mortality rate was zero.

### Estimation of initial lesion volume

A group of 5 male Sprague Dawley rats (6 weeks old, mean weight 193±4 g) were given impact injuries at the exposed 10th thoracic spinal segment as described above. At 15 min after injury, the animals were killed by an overdose of inhaled isoflurane and the spinal cords removed and immersion fixed in 4% paraformaldehyde solution for 24 hours. A 10 mm length of spinal cord centred on the middle of the lesion site was removed and embedded in a 4% agar solution. Serial sections (100 µm thick) were cut in a vibrating microtome (Leica VT1000S), mounted on glass slides and images captured at 4× magnification with a digital camera (Olympus DP70) attached to a compound microscope (Olympus BX50). At 15 min after injury, sections through the centre of the lesion contained numerous haemorrhage spots and visible tissue damage mainly in the central grey matter and occasionally in the dorsal column white matter. The lateral and ventral white matter appeared largely intact with very few haemorrhage spots or physical damage. In each serial section, the area of tissue occupied by the lesion (visibly damaged tissue and haemorrhage, see Fig. 1D) was outlined and measured (ImageJ, NIH). The volume of tissue occupied by the lesion between successive pairs of serial sections was calculated from the area measurements and the known distance between the sections (100 µm) and summed to determine the total lesion volume.

### Immunocytochemistry

At different times after spinal cord injury(2-, 24-hours, 4 days, 4- or 10-weeks), the injured rats and age-matched sham controls (n = 3–4 per group) were terminally anaesthetised. Brains were perfuse-fixed with a heparinised phosphate buffered saline pre-wash (50 ml) followed by a 4% paraformaldehyde solution (200 mL) in 0.1 M phosphate buffer (pH 7.3) delivered at a flow rate of 8 mL/min/100 g body weight (80% of the estimated cardiac output for adult rats). The spinal cords were removed and post-fixed overnight in the same fixative at 4°C. A 10 mm segment of spinal cord encompassing the site of the lesion (or an equivalent segment from sham operated animals) was removed and post-fixed in Bouin's fixative for 24 hours. After serial washes in graded ethanol and clearing in chloroform, the tissue was paraffin embedded in 55°C melting wax and serial 5 µm transverse sections cut. Consecutive sections were placed on glass microscopic slides (10 per slide). Every 10th slide was routinely stained with haematoxylin and eosin (H&E) for histological examination. Remaining slides were used for immunocytochemical analysis using a range of antibodies for different neuronal markers: NSE (DAKO, diluted 1∶200), NeuN (Chemicon, diluted 1∶100) and PGP9.5 (DAKO, diluted 1∶100). All antibody dilutions were made in PBS/Tween20, pH 7.4. Every 10th slide was stained for each marker by incubation in primary antibodies overnight at 4°C followed by incubation with secondary antibodies: swine anti-rabbit or rabbit anti-mouse (both from DAKO in 1∶200 dilutions in PBS/Tween20) and corresponding tertiary antibodies: rabbit PAP or mouse PAP (DAKO, 1∶200 dilution in PBS/Tween20). The reaction product was developed with DAKO DAB staining kit, dehydrated and mounted with DPX. Slides that were incubated without the specific antibodies were used as controls and these always appeared blank. Immunostained slides were used for the quantitative analysis as described below.

### Image analysis of tissue sections

Hematoxylin and eosin or immunocytochemically stained sections were used for quantitative analysis of tissue following SCI and compared to age-matched sham operated animals. All images were captured with an Olympus DP-70 digital camera attached to an Olympus BX50 microscope. Images were captured using the same camera, lens and exposure conditions. All images were analysed using ImagePro Plus software (version 4.5.1.22). One representative section on every 10th slide corresponding to every 500 µm along the cord was used for each cell marker. Although it is not possible to conduct proper blind experiments, as injured cords are immediately identifiable, every attempt was made to remove any bias by mixing sections from different age groups for both staining and counting. All NSE, PGP9.5 or NeuN positive neurons within the grey matter of the spinal cords were counted manually.

### Stereological estimations of axon numbers

Uninjured rats along with injured animals at 24 hours, 1- 4- and 10-weeks after injury (n = 3 per group) were killed with inhalation of isoflurane (>10%). Animals were immediately perfused through the aorta with cold heparinised phosphate buffered saline (pH 7.3) for 90 s and then for 10 min with a mixture of paraformaldehyde and glutaraldehyde (1.5% each) in 0.1 M phosphate buffer (pH 7.3). A 10 mm spinal cord segment centred on the injury site was removed and left in fixative for 2 hours at 4°C. In control animals the equivalent segment of the cord was removed. Tissue was washed in 0.1 M phosphate buffer, embedded in 4% Agar, sectioned into 1 mm slices using a vibrating microtome and left overnight in fixative. The 1 mm spinal cord sections were processed for conventional electron microscopy. Tissue was rinsed in 0.1 M cacodylate buffer, treated with 2% osmium for 1 hour followed by uranyl acetate (in 70% acetone) for 30 min, dehydrated in increasing concentrations of acetone and flat embedded in Epon. Agar was left around the tissue in order to identify and orientate each piece of spinal cord during embedding. Sections of 0.5 µm thickness were cut from each 1 mm spinal slice using a Reichart ultramicrotome and stained with methylene blue. The total number of myelinated axons in the cord was estimated in the 0.5 µm thick sections using a motorised Stereology system with Stereoinvestigator 7.0 software. The dorsal column excluding the corticospinal tract at the base of the dorsal column was outlined and the total number of myelinated axon profiles within this area estimated using 50 counting frames at 2000 magnification. In a similar manner the number of axons in ventrolateral tracts was estimated. Only myelinated axon profiles above 0.5 µm and below 10 µm diameter were included in counts. 0.5 µm was the lower limit to clearly identify myelinated axons at this magnification and 10 µm was set as the upper limit to exclude axons with severe pathology (in control tissue the largest axons were 6–7 µm in diameter). Many axon profiles at 24 hours after injury showed acute pathology with swollen axons (15–25 µm diameter) and their myelin sheaths detached from the axon and these were not included in counts.

### Western blotting

Following behavioural analysis (see below) spinal segments were collected from sham operated and spinal cord injury animals at 24 hours, 4 days, 4- and 10-weeks after surgery (n = 6 at each time). Animals were killed by an overdose of inhaled isoflurane followed by opening of the chest cavity and transection of the heart. The entire spinal cord was removed and rinsed in PBS (phosphate-buffered saline, pH 7.4) containing protease inhibitor (Complete Mini, Roche). A 10 mm segment centred on the injury site was collected, immediately immersed into a pre-weighed tube containing PBS with protease inhibitor, weighed and kept at −20°C. The following day the spinal segments were homogenised in an all glass homogeniser and protein extraction was performed using the ProteoExtract Subcellular Proteome Extraction Kit (Calbiochem) according to the manufacturer's instructions. All samples were centrifuged for 5 min at 10000 g prior to all analysis steps. Protein content was measured using the standard Bradford Assay [14]. An appropriate amount of total protein that was constant for each marker (range 0.07–1.0 µg) was loaded into each well (30 µL wells, 12% HCl-Tris Pre-Cast gels, BioRad) and separated using electrophoresis (Criterion System, BioRad). The proteins were transferred onto a PVDF membrane (BioRad) using wet transfer. The PVDF membrane was blocked overnight at 4°C in a solution containing 50% soymilk, 48.8% tris-buffered saline (TBS) and 0.2% Tween. The membranes were incubated with primary antibodies against PGP9.5 (DAKO, 1∶500), NSE (Chemicon, 1∶5000), GFAP (DAKO, 1∶2000), CNPase (Sigma, 1∶500) and β-actin (Sapphire BioSciences, 1∶3000) in blocking solution (25% soymilk in PBS and 0.1% Tween) for 1.5 hours at room temperature followed by 3×10 min washes in TBS. The membrane was incubated for 1.5 hours with HRP conjugated anti- mouse (1∶4000, Chemicon) or HRP conjugated anti-rabbit secondary antibody (1∶4000, Amersham Biosciences). Following further washes the antibody complexes were visualised and the levels of relative protein levels quantified using chemiluminesence (ECL kit, Amersham Biosciences) and Kodak Image Station. The antibody concentration and the amount of protein loaded were optimised for each marker in order to ensure that detection was within the linear range and that the primary antibody was present in excess. Each gel contained two concentrations of a reference sample and all gels were run in duplicate. The relative density of each band was compared to the mean density of the sham-operated group and results presented as mean ± SEM.

### Behavioural methods

#### BBB Open Field Locomotor Score

The Basso-Beattie-Bresnehan (BBB) locomotor score was used to rate hindlimb movements following spinal cord injury as previously described [15]. BBB scoring was conducted in an open field and a score of 21 was considered normal and a score of zero indicated no hindlimb movement.

Ledged Beam: Skilled motor co-ordination was measured using the tapered ledged beam [16]. The beam consisted of a 1.35 m long central section, which tapered from 5.5 cm to 1.5 cm from one end to the other. Underhanging ledges 2 cm in width were located 2 cm below the upper surface of the beam (Dragonfly Research and Development Inc., West Virginia, USA). Automatic counters recorded contact with the ledge each time it was depressed by a foot. Prior to spinal cord injury rats were trained to walk the length of the beam from the wide to the narrow end where a black box was located. Naïve rats were able to walk the length of the beam without using the underhanging ledges for support. Rats were tested from 2 weeks after injury at weekly intervals in separate trials for 4 or 10 weeks. Spinal cord injured rats were tested only if they were able to use their limbs to support their own weight clear of the ledged beam surface.

#### Random Rung Horizontal Ladder

The random rung ladder consisted of a 1.5 m long tunnel containing a ladder with rungs 0.5 cm in diameter and 1 cm apart. Rungs were randomly removed to make gaps of 1 to 4 cm. This test requires the rat to be able to coordinate hindlimb movements with forelimb movements in order that the hindlimbs do not slip through the gaps between the rungs. Prior to spinal cord injury or sham surgery, rats were trained to walk the length of the ladder and were then tested weekly over 2 trials for 4 or 10 weeks. Trials were videotaped and later analysed by an experimenter blinded to treatment condition. The number of hindlimb foot slips were counted and averaged over the 2 trials.

DigiGait™ Analysis: Footprint analysis was conducted using a computerised digital footprint analysis system (DigiGait™, Mouse Specifics Inc., MA. USA). Rats were tested once only at experimental endpoints of 4 or 10 weeks. Only rats that could support their own weight were tested. Rats were accustomed to the treadmill at gradually increasing speeds prior to recording at least 4 complete step cycles at a speed of 10 cm/s. Rats were placed in the Perspex chamber on the motorised transparent treadmill and the movement of the paws was captured from the ventral aspect using a high-speed digital video recorder (Basler A602 camera 150 fps) mounted underneath. Various indices of gait were then automatically calculated by the DigiGait™ analysis software from digitised images of paw contact with the treadmill.

### Statistical analyses

All data are presented as mean ± standard deviation (SD) or ± standard error of the mean (SEM). Behavioural data were analysed using analysis of variance (ANOVA) and individual differences were determined using Bonferroni's post-test.

## Results

### Volume of initial injury

The mean velocity of the impactor tip (Fig. 1A,B) at the time of contact with the dura mater was 0.96 m/s (±0.003 SD, n = 34) with an average tissue penetration depth of 2.01 mm (±0.05 SD, n = 34) from the surface of the dorsal dura mater. The mean impact force, taking into account the degree of tissue displacement, was 28.5 Newtons (±0.87 SD, n = 34), see Fig. 1C. The mean lesion volume at 15 min after injury, calculated from lesion area measurements in 100 µm thick serial sections (Fig. 1D) spanning the entire injury site, was 1.92±0.23 mm^3^ (SD, n = 5) within a range of 1.52 to 2.10 mm^3^ (Fig. 1E).

### General progression of injury

**Figure 2:**
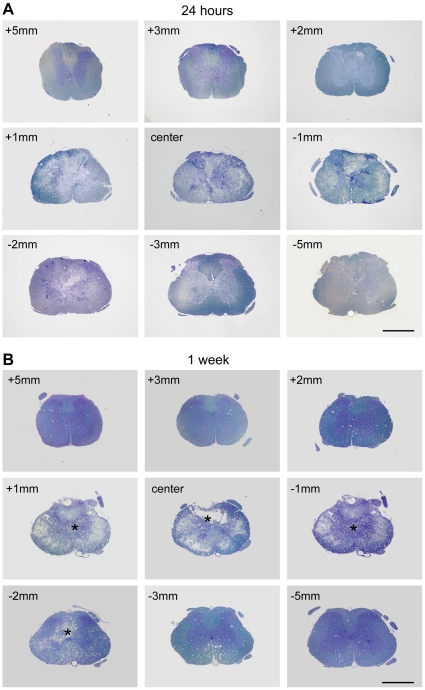
Morphology of injury at 24 hours and 1 week. Methylene blue stained semi-thin sections (0.5 µm) of spinal cords at 24 hours (**A**) and 1 week (**B**) after injury, from 5 mm rostral (+) to 5 mm caudal (−) of the centre of the injury site. At 24 hours damage was apparent within 2 to 3 mm of the centre of the injury site in both grey and white matter. No macrophages were visible in the tissue. At 1 week tissue was being cleared by numerous macrophages (*) that had infiltrated the tissue and the early stages of cyst formation can been seen at the centre of the injury. Scale bars are 1 mm.

**Figure 3:**
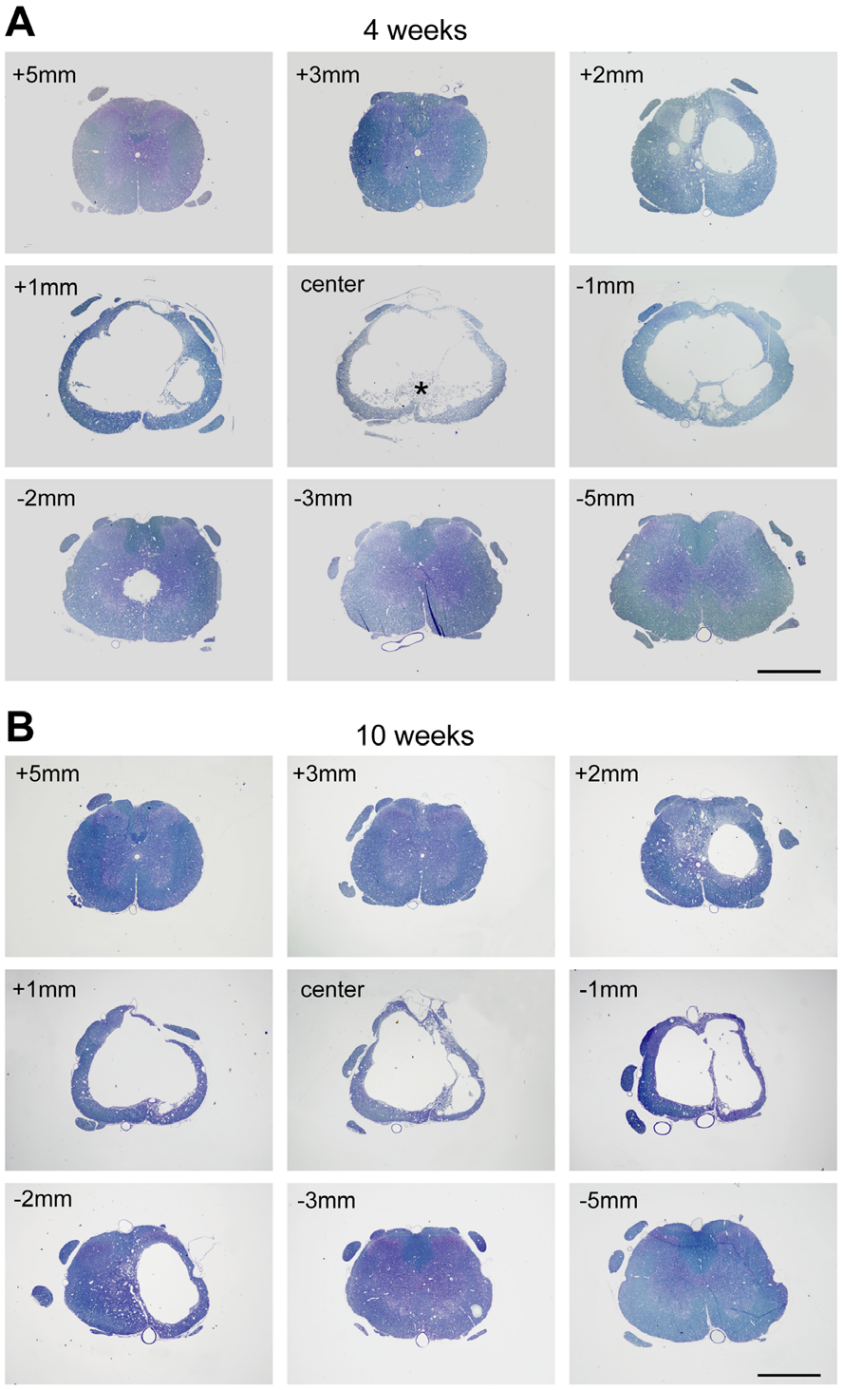
Morphology of injury at 4 and 10 weeks. Methylene blue stained semi-thin sections (0.5 µm) of spinal cords at 4 weeks (**A**) and 10 weeks (**B**) after injury, from 5 mm rostral (+) to 5 mm caudal (−) of the centre of the injury site. At 4 weeks a large cystic cavity was visible within the cord extending several millimetres along the cord. A small number of macrophages were present in the centre of the injury (*). A similar cystic cavity was present at 10 weeks, however, no macrophages were seen in the cord. Scale bars are 1 mm.

At 15 min after injury, tissue damage and haemorrhages were largely confined to the central grey matter with little visible damage in the surrounding white matter (Fig. 1D). By 24 hours, there was further disruption involving most of the grey matter at the site of the lesion and extending into the adjacent white matter (Fig. 2A). By one week, macrophages were present in the injury site and there was evidence of clearance of damaged tissue (see stars in Fig. 2B). By 4 weeks, there had been further clearance of damaged tissue with the formation of a large fluid filled cystic cavity in the centre of the spinal cord with only a thin rim of white matter remaining underneath the pial surface (Fig. 3A). By 10 weeks, there was no further visible expansion of the cystic cavity and only a few macrophages remained in the injury site (Fig. 3B). Well above the injury site (+10 mm), there was a pronounced loss of axons in the medial part of the dorsal columns, but no loss in the ventral parts of the dorsal column (not illustrated). In contrast, well below the injury site (−10 mm) there was no loss of axons in the medial parts of the dorsal column but a prominent loss in the ventral parts of the dorsal column (not illustrated).

**Figure 4:**
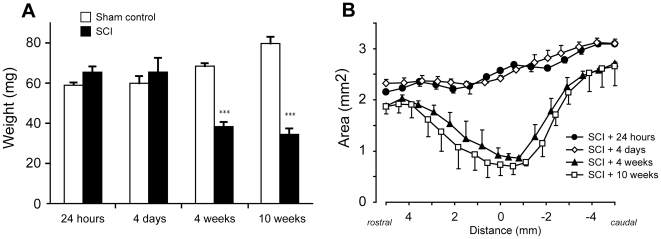
Measurement of spinal cord weight and area. **A**) Weights (mg) of 10 mm spinal cord segments centred on the injury site. There was a significant reduction in weight compared to sham controls at 4 and 10 weeks after injury (*p<*0.001, ***). In injured animals, there was a significant decrease in weight between 4 days and 4 weeks after injury (*p<*0.01, **), but no further weight loss between 4 and 10 weeks after injury. Data are mean ± SEM, n = 6 for each group. **B**) Areas of spinal cord measured in H&E stained serial sections from 5 mm rostral to 5 mm caudal of the centre of the injury site at 24 hours, 4 days, 4 weeks and 10 weeks after injury. There was little difference in spinal cord area between 24 hours and 4 days, but a large reduction in area between 4 days and 4 weeks. There were no further reductions in area between 4 weeks and 10 weeks after injury. Data are mean ± SEM, n = 6 for each group.

The tissue weights of 10 mm segments of spinal cord at the T10 level from sham-operated rats increased by approximately 35% between 24 hours and 10 weeks post-operation (*p*<0.001, Fig. 4A) reflecting the natural growth of the animals. However in the spinal-injured animals, the weights of 10 mm segments of spinal cord centred about the lesion site were approximately 45% lower than their age matched sham controls at 4 and 10 weeks after injury (*p*<0.001) but not significantly different from controls at 24 hours and 4 days. Similarly, measurements made in H&E stained serial sections showed little difference in tissue cross-sectional area between 24 hours and 4 days, but a marked reduction in tissue cross-sectional area was apparent at 4 and 10 weeks (Fig. 4B). This loss was greatest at the centre of the injury site and tapered off towards both the rostral and caudal ends of the segment. There was no significant difference in the amount of tissue loss between 4 and 10 weeks. This estimation based on H&E sections does not differentiate between damaged and undamaged tissue, rather it reflects the progression of cellular clearance following injury.

### Progression of injury based on neuronal counts

**Figure 5:**
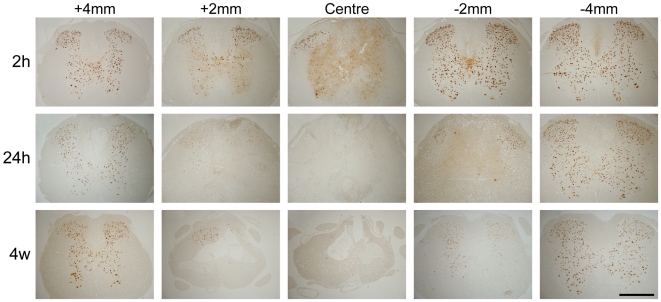
NeuN immunoreactivity in tissue sections. NeuN positive nuclei in spinal cord at 2-, 24-hours and 4 weeks after injury. At 2 hours (2 h), a small number of neurons were visible at the centre of the injury site located mainly in the dorsal horns. By 24 hours (24 h) there had been further loss of neurons up to 2 mm either side of the centre of the injury site. NeuN profiles at 4 weeks (4w) were similar to that at 24 hours indicating that the loss of grey matter neurons occurs within the first 24 hours (see also Fig. 6).

**Figure 6:**
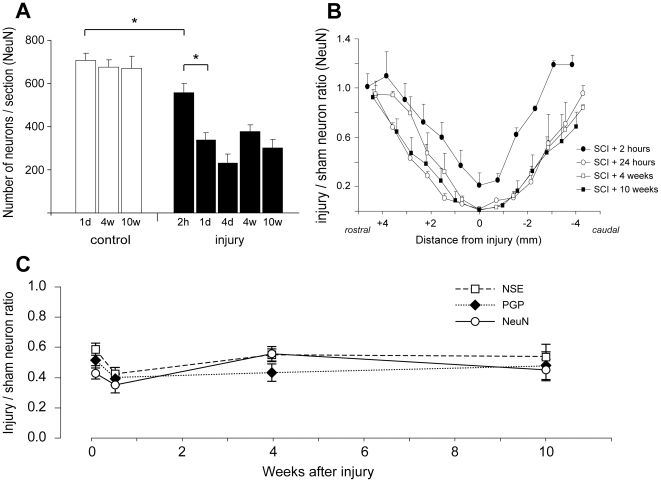
Counts of neurons in spinal cords. **A**) Number of NeuN immunoreactive nuclei per section (mean ± SEM) in 10 mm segments centred on the injury site at different times after surgery (n = 3–4 animals for each group). For each animal, the average of 12–14 sections was determined. In sham-operated animals (open bars), there was little difference in number of neurons between 24 hours, 4 weeks and 10 weeks after surgery. In the injured animals, the number of neurons was significantly lower at 2 hours compared to sham controls (*p*<0.05, *) and was further reduced at 24 h (*p*<0.05, *). At 24 hours, 4 days, 4 weeks and 10 weeks, the number of neurons was significantly lower than in age matched sham controls (*p*<0.01) and compared to 2 h after injury (*p*<0.05). There was no significant change in the number of neurons from 24 hours to 10 weeks after injury. 2 h = 2 hours, 1d = 24 hours, 4d = 4 days, 4w = 4 weeks, 10w = 10 weeks. **B**) Number of NeuN neurons per section (mean ± SEM) in injured animals normalised to control animals at different distances from injury site (0 mm). At 2 hours after injury, the number of neurons was higher at each point along the cord compared to all later times after injury. There was little difference in neuron numbers at any point along the cord from 24 hours to 10 weeks after injury. Note the similar pattern of neuron loss rostral and caudal to the injury site. **C**) Number of grey matter neurons (immunoreactive for PGP9.5, NSE or NeuN) per section normalised to sham operated control animals in 10 mm segments centred on the injury site at different times after surgery. The similar neuron numbers from 24 hours to 10 weeks after injury indicate that the loss of grey matter neurons occurs within the first 24 hours. Data are mean ± SEM, n = 3–4 for each group.

Neuronal profiles, immunoreactive for individual cellular markers (NeuN, PGP9.5 or NSE), were counted manually in 5 µm thick paraffin sections at every 500 µm throughout the 10 mm spinal cord segments centred on the injury site. Representative tissue sections stained for NeuN are illustrated in Fig. 5 at different distances from the injury site at 2-, 24-hours and 4 weeks after injury. In each cord a minimum of 12 sections was used for each marker. Data for NeuN positive neurons are presented in Figs. 6A and 6B. In sham operated animals there was little difference in the mean number of neurons per section between 24 hours, 4 weeks and 10 weeks after surgery (707±35, 677±34 and 670±55, respectively, see Fig. 6A). In injured animals the number of NeuN positive neurons was significantly lower at 2 hours (558±41, *p*<0.05) compared to sham controls. At 24 hours, 4 days, 4 weeks and 10 weeks after injury the mean number of NeuN positive neurons per section (338±34, 232±42, 377±32 and 301±41, respectively) were significantly lower compared to sham operated control animals and also compared to 2 hours after the injury (*p*<0.05). However, there were no significant differences between 24 hours, 4 days, 4- or 10-weeks. Fig. 6B illustrates the numbers of NeuN positive neurons in serial sections along the length of the lesion at each time after injury studied showing that in the middle of the injury the number of neurons at 2 hours after injury was about 21% of sham controls whereas at all later time points the number of neurons was close to zero and that the number of neurons was symmetrical on either side of the injury at all times after the injury. There were no significant differences between injured and sham animals or the ratios of neuronal profiles stained for NeuN, NSE or PGP9.5 between 24 hours, 4 days, 4 weeks or 10 weeks after the injury (Fig. 6C).

### Stereological estimations of axon profile numbers

**Figure 7:**
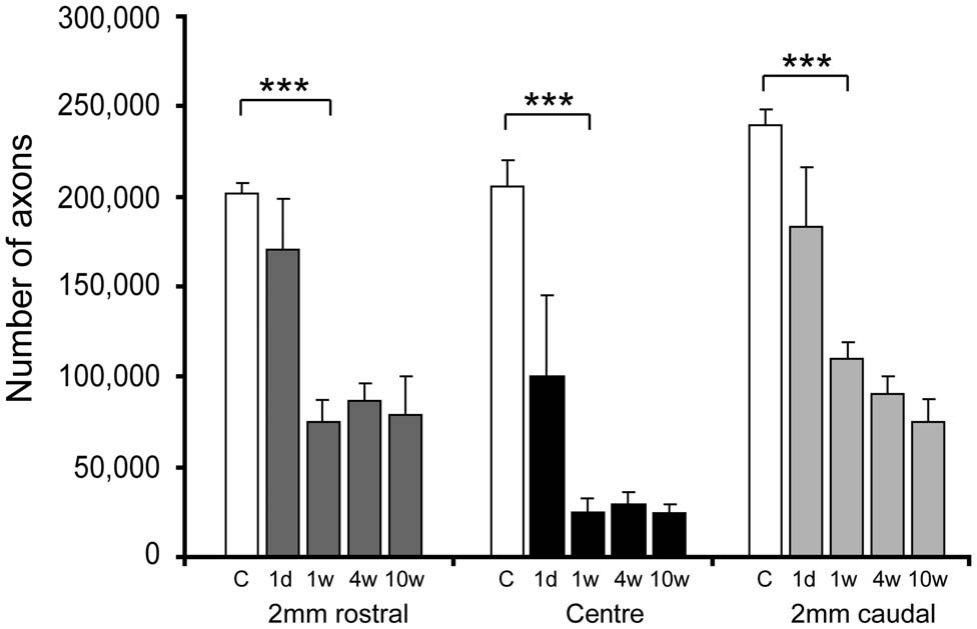
Myelinated axons in spinal cords. Stereological estimation of numbers of myelinated axons at the centre, 2 mm rostral and 2 mm caudal of injury site in control and injured animals. At 24 hours after injury, the number of axons at the centre of the injury site was reduced to 50% of control animals (open bars) and at 1–10 weeks was further reduced to 12–15% of controls. At 2 mm rostral to the centre of the injury site, the number of the number of axons was 85% of control animals at 24 hours after injury and 37–43% of controls at 1–10 weeks. At 2 mm caudal to the centre of the injury site, the number of axons was about 80% of control animals at 24 hours after injury and 30–45% of controls at 1–10 weeks. C  =  sham control, 1d = 24 hours, 1w = 1 week, 4w = 4 weeks, 10w = 10 weeks. Data are mean ± SEM, n = 3 for each group. *** = *p<*0.001.

The number of myelinated axon profiles was counted in plastic semi-thin cross-sections at the centre, 2 mm rostral and 2 mm caudal to the injury site at different times after injury using stereology-based microscopy (Fig. 7). The number of axons in control animals was 200,000–240,000 in the lower thoracic (T10) spinal cord. At 24 hours after injury, the number of axons at the centre of the injury site was approximately 100,000 (or 50% of age-matched, control animals, *p*<0.001) and at 1-, 4- and 10-weeks the number of axons was 24,000–31,000 (13–15% of age-matched controls, *p*<0.001). At 2 mm rostral to the injury site, the number of axons was 170,000 at 24 hours after injury (not significantly different from control animals) whereas at 1 to 10 weeks the number of axons was 76,000–86,000 (around 40% of control animals, *p*<0.001) and was also significantly lower than 24-hour animals (*p*<0.05). At 2 mm caudal to the injury site, the number of axons at 24 hours after injury was 183,000 (77% of control animals) and at 1-, 4- and 10-weeks was 75,000–110,000 axons (30–45% of control animals, *p*<0.001 compared with controls).

### Progression of injury assessed by Western blot analysis

**Figure 8:**
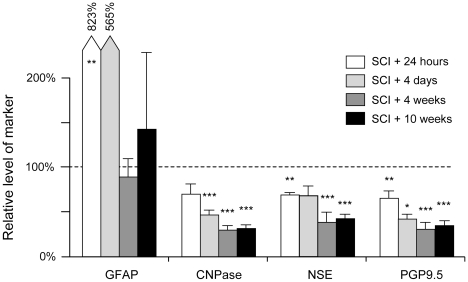
Western blot analysis. Analysis for GFAP, CNPase, NSE and PGP9.5 in 10 mm spinal cord segments centred on the injury site. Constant amount of protein (range 0.07–1.0 µg) for each marker was loaded into each well. All data are expressed as percentage change (± SEM) compared to sham operated age-matched control tissue. GFAP (specific for astrocytes) showed very large increases at 24 hours and 4 days after injury, which is a reflection of the activation of astrocytes in response to injury. GFAP levels were not significantly different from controls at 4 and 10 weeks after injury. In contrast, the levels of the NSE and PGP9.5 (specific for neurons) and CNPase (specific for oligodendrocytes) were reduced to 70% of controls at 24 hours after injury and to 30–40% up to 4 weeks. There were no further reductions up to 10 weeks after injury. Data are mean ± SEM, n = 6 for each group. Probability of significant difference compared to age matched sham operated controls: * = *p*<0.05, ** = *p*<0.01, *** = *p*<0.001.

The progression of injury was also estimated by semi-quantitative Western blotting using several cellular markers: GFAP for activated astrocytes, CNPase for white matter oligodendrocytes and NSE and PGP9.5 for neurons (Fig. 8). GFAP content increased several fold at 24 hours and 4 days after SCI compared to age-matched sham operated controls, but returned close to control levels at 4 and 10 weeks after trauma. The other markers (CNPase, NSE and PGP9.5) were all lower in SCI animals compared to sham-operated controls (Fig. 8). 24 hours after injury all 3 markers had declined to about 70% of controls. CNPase declined further to about 45% of controls by 4 days (*p*<0.001) and to 30% (*p*<0.05) at 4 and 10 weeks after SCI (*p*<0.05 compared to 4 days after injury). There was no significant difference in CNPase content between 4 and 10 weeks post-trauma. NSE content was 70% of control at 24 hours (*p<*0.01) and 4 days, but declined to under 40% of control levels at 4 and 10 weeks (*p*<0.001). PGP9.5 content was also significantly reduced from about 70% of control levels at 24 hours (*p*<0.01) to 40% at 4 days (*p*<0.05) and 30% at 4 (*p*<0.001) and 10 weeks.

### Behavioural assessment

**Figure 9:**
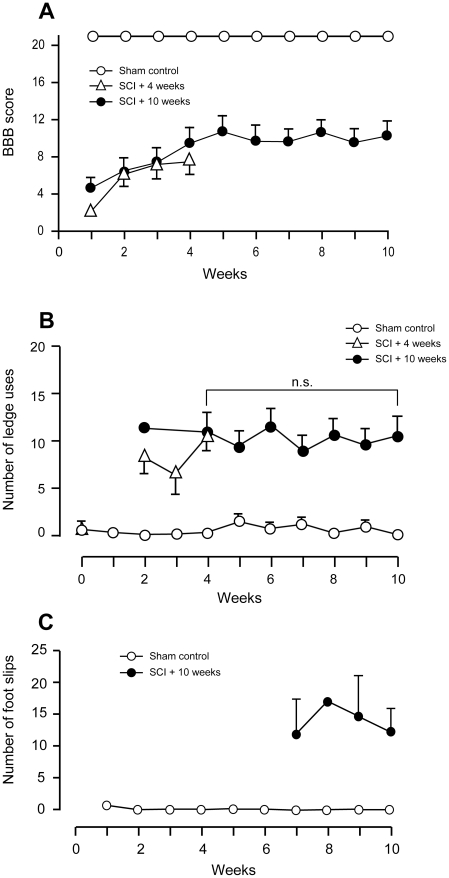
Behavioural testing. **A**) BBB scores for sham operated (open circles, n = 8) and spinal cord injured animals at 4 weeks (open triangles, n = 4) and 10 weeks (filled circles, n = 8–13) after injury. There was an improvement in BBB scores from 1 week up to 4 weeks after injury, but no further improvement up to 10 weeks. At all times after injury, the scores for the injured animals were significantly lower than for age-matched sham controls (*p*<0.001). Data are mean ± SEM. **B**) Number of errors (uses of the ledge by hindlimbs) in the ledged beam test by sham control (open circles, n = 8) and spinal cord injured rats at 4 weeks (open triangles, n = 4) or 10 weeks (filled circles, n = 8–13). The number of errors by the injured animals was significantly greater than sham controls at all time points (*p*<0.001). Data are mean± SEM. **C**) Number of errors (hindlimb foot slips) in the random rung ladder beam test in sham control (open circles, n = 4) and spinal cord injured rats at 10 weeks (filled circles, n = 4). The spinal cord injured animals made significantly more errors than age matched sham controls at each time point (*p*<0.001). Data are mean ± SEM.

Spinal cord injured rats showed very few hindlimb movements up to one week after injury and scored only 2–3 on the BBB scale compared with normal scores of 21 for sham operated rats (Fig. 9A). A gradual improvement in BBB scores occurred after 1 week for both SCI groups reaching 8 (±1 SEM) at 4 weeks (*p<*0.05 compared to 1 week after injury). This score represents animals on the border of producing stepping movements with no weight support to some weight-supported stepping movements. There was no further improvement in BBB scores from 4 to 10 weeks after injury (Fig. 9A).

From 2 weeks following injury in the ledged beam test (the earliest when they could be tested) the spinal cord injured rats made significantly more uses of the ledges (11.8±2.8) with their hindlimbs than sham operated animals, who made no errors. In contrast to the BBB scores, there was no improvement in performance at any time after injury (Fig. 9B). Similarly, in the random rung ladder test, spinal injured rats made many hindlimb slips through the missing rungs (12.6±1.7) whereas sham animals made no slips at all. It was only possible to perform the random rung ladder beam test at later times when the animals were able to bear their body weight. There was no improvement in performance during 7 to 10 weeks after injury (Fig. 9C).

#### DigiGait™ analysis

21 gait parameters were measured using a computerised footprint analysis system (DigiGait™). Multiple changes in gait parameters in both the hindlimbs and in the forelimbs were found in the spinal injured animals. Changes in the forelimbs probably reflect compensatory adjustments for the hindlimb deficits. The majority of the gait parameters did not change significantly between 4 to 10 weeks after injury suggesting no improvement in gait performance with time. Only three inter-dependant hindlimb gait parameters were significantly different (*p*<0.05) at 10 weeks compared with 4 weeks after injury: % of stride in swing phase (20.4±1.3 and 14.5±1.7, respectively), % of stride in stance phase (79.6±1.3 and 85.0±1.4, respectively) and the ratio of time spent in stance and swing phase (4.2±0.3 and 6.1±0.6, respectively). Although suggestive of improvement, observation of high-speed video recordings indicated that this was not the case. While the percentage of time hindlimbs were in the swing phase was significantly increased in 10 week injury animals, the videos clearly showed that this was largely due to an increase in the amount of time required for the rats to swing the hindlimb through a single step cycle and place the paw back on the treadmill. The stance phase of the hindlimbs was accordingly reduced as the treadmill moved on and concomitantly the forelimbs spent significantly less time in swing and more time in stance while the hindlimbs caught up. An increased stride frequency of the forelimbs and reduced step angle was observed between 4 and 10 weeks after injury, which appeared to be compensatory changes to maintain balance. The hindlimb shared stance time (the amount of time the hindlimbs were both in stance together), was significantly decreased in the spinal injured animals compared to sham operated controls, indicating an improvement in the alternating movement of the hindlimbs, but paw sequence or percentage alternation between forelimbs and hindlimbs was not improved.

There was no change in percentage of stride and stance in braking phase, percentage of stride and stance in propulsion phase, stance duration and stance factor, or the ratio of left to right stance durations between 4 and 10 weeks after injury or between injured and sham operated controls at either time for these parameters. Gait parameters in the sham-operated animals did not change over time with the exception of the maximum paw area at peak stance, this being due to the marked increase in foot size and weight of the animals over time.

## Discussion

In this study we have used a combination of complementary techniques to measure the total lesion volume, areas of residual grey and white matter, numbers of surviving neurons, myelinated axons and behavioural performance at different times after spinal contusion injury. This has provided quantitative measurements of the relative contributions to the processes of secondary damage from cell death, axonal loss and tissue clearance following injury.

### Contusion injuries of the spinal cord

Human spinal cord injuries are extremely variable in their extent and pathology [17], therefore a reproducible method of producing spinal trauma is required, otherwise potentially useful therapies will be lost in the haze of variability. A complete or partial transection of the spinal cord is widely used as a method for producing spinal cord injury e.g. [18]–[21], which is appropriate for questions concerning the response of neurons to axotomy and for studies of regeneration. However it is less appropriate for assessing potential therapies, because simple axotomy is rare in human spinal cord injuries and may have quite different effects on the vascular supply compared to compression injuries. Closed injuries to the brain [22] or spinal cord [23] approximate better to what happens in patients, but are subject to such variability [24] that they are of limited value for initial quantitative assessment of therapies as they have been shown to produce injuries that vary in volume by 20 fold [24]. For qualitative studies of pathological changes following injury, this may not be a serious limitation, but quantitative assessment of potential therapies would require large numbers of animals (e.g. n = 70 to detect a 25% reduction in the size of injury.

Contusion models of spinal cord injury date back to the original weight drop studies of Allen [25]. In the past 25 years several more sophisticated versions of this technique have been developed that allow various parameters of the contusion device to be measured and used to select which animals should be included or excluded in a study, for example, the New York impactor [26] also known as MACSIS, Multicenter Animal Spinal Cord Injury Study [27]. Other weight drop methods developed include the one first described by Wrathall et al. [28] in which a weight strikes a rod placed in contact with the dorsal surface of the spinal cord. An alternative approach for inducing controlled spinal cord contusion was the development of an electromagnetic device [29], [30] and more recently motor driven impactors [12], [13] and Infinite Horizons impactor [31]. The size and associated variance of the lesions produced by these devices at the time of impact is not clear from the published literature, but the latter seems likely to be significant since some studies report the use of exclusion criteria to remove animals from a study that have injuries outside the expected parameters [32], [33]. Most of the assessments of the performance seem to have relied on measuring various parameters of impact (e.g. force, velocity and tissue displacement), estimation of injury size days or weeks after injury and assessment of behaviour usually using the BBB [15] observational scale. These electronic devices typically provide control over the velocity of the impactor rod or the force of impact that is applied. However Kim et al. [34] recently demonstrated that the extent and severity of tissue damage produced by the most commonly used of these impactors is determined primarily by the depth to which the impactor rod penetrates into the spinal cord and not by its velocity, which is often used as a key parameter for standardising the impacts. Furthermore, the impact force from a moving rod striking a stationary surface is a function of the mass of the impactor rod, it's velocity and the resistance to deformation (compliance) of the surface being impacted. Thus differences in tissue compliance will result in different depths of penetration for both weight drop and force feedback type impactor devices.

To minimise these potential sources of variance, a computer controlled impactor system based on a linear motor device previously described [12], [13] was constructed and used in the present study. This device allows precise control over multiple parameters of the impact: (a) the incident angle of the impactor rod relative to the surface being struck, (b) the acceleration rate and velocity of the impactor rod, (c) the depth of penetration of the impactor tip into the spinal cord, (d) the length of time the tissue remains compressed and (e) the rate of withdrawal of the impactor rod, which prevents any rebound strikes. Quantitative estimations of initial lesion size and reproducibility using this type of motorised impactor have not previously been published. The key features of the device used in the present study are that the depth of tip penetration into the spinal cord is preset and controlled, the incident angle of the impactor rod is not limited to the vertical and can be adjusted to deliver the impact at right angles to the surface being struck (which is particularly useful for producing consistent brain contusion injuries) and the vertebral column is stabilised by clamps. The results presented in this paper show that this device produces consistent low variance injuries of spinal cord, when care is taken to adequately stabilise the vertebral column with clamps onto adjacent vertebrae (see Fig. 1B). As a consequence, very few animals (<2%) were excluded from this study. The same device also produces very low variance lesions when used for traumatic brain injury studies (<10%, R. Medcalf, personal communication).

### Measurement of injury size

Methods of estimating the size of experimentally produced spinal cord injuries have varied from qualitative scoring of histological sections [35] to morphometric measurements of serial sections at different points through the lesion [36]–[38]. However, some authors have used only measurements of cross sectional area at the centre of the lesion to estimate its size e.g. [32], [39] or used sections at the limit of the lesion and in the centre of the lesion to estimate lesion volume e.g. [40], neither approach is likely to be adequate since the lesions may not be symmetrical. Furthermore, morphological measurements of the volumes of the lesions produced by the earlier impactor models do not appear to have been made shortly after the impact. Instead, the measured performance parameters of the impactor device have been used as inclusion/exclusion criteria for which animals should be retained in a study. Usually the number of animals discarded is not recorded, but in one instance nearly 50% of animals in the severe injury group were not used [33] which gives cause for concern about appropriate animal use and bias. A more detailed critique of spinal cord injury methods will be published separately (Habgood, in preparation).

### Morphological changes following spinal cord injury

In this study we have used a combination of morphological and biochemical methods to assess lesion size and changes in individual constituents of the surviving tissue at different times following injury. The results from the diverse measures used (morphological appearance on sections, immunocytochemistry and western blotting of neuronal markers) indicate that loss of grey matter occurs only in the first 24 hours after injury, whereas white matter loss, the assessment of which included systematic counting of axonal profiles in semi thin sections, continues up to about 7 days post injury. The early loss of grey matter in and around the lesion site has also been reported by others [41], [42] and may be initiated by disruption of the vascular supply to this tissue (see below).

One of the most striking features of the spinal cords collected soon after injury was the apparently normal appearance of the white matter (see Fig. 1D, 2A and also [39]). At 15 min after injury, visible tissue damage was largely confined to and occupied most of the grey matter at the centre of the injury site and consisted mainly of tissue compression, shearing, ruptured blood vessels and punctate haemorrhages (Fig. 1D and [13]). This is despite the impact being delivered onto the dorsal white matter. With increasing distance rostral or caudal to the lesion centre, the extent of visible grey matter damage tapered off rapidly and at only 2 mm from the centre of the lesion the spinal cord appeared visibly undamaged (see Fig. 1E). However, published MRI images from a study using a similar device with a slightly shallower (1.7 mm) penetration depth [13] have revealed the presence of edema in both the central grey matter and adjacent white matter for up to 4 mm from the centre of the lesion suggesting that undamaged tissue adjacent to the injury site is at risk of secondary damage. The initial absence of visible damage in the surrounding white matter suggests that this tissue may offer much greater resistance to deformation than does the deeper grey matter.

Away from the lesion centre the loss of myelinated axons was less pronounced (Fig. 7). This more protracted time course of white matter damage, compared to grey matter, suggests that white matter should be a key target for initial therapeutic treatments and that there is a window of opportunity of several days during which agents could be administered to help support white matter survival.

Clearance of damaged tissue was not evident in cross sections through the centre of the injury until 1 week post trauma (see Figs. 2, 3, 4B) and a significant reduction in cord weight was not evident until 4 weeks after injury (Fig. 4A). The fact that there was significant clearance of tissue between 1–4 weeks after injury, whereas the loss of grey matter neurons and white matter axons was complete by 24 hours and 1 week respectively, indicates that ongoing tissue clearance should not be interpreted as evidence of further secondary loss of either neurons or white matter tracts. Thus simple measurements of remaining tissue areas or volumes are not appropriate measurements for estimating loss of grey or white matter.

### Western blot analysis

Most histological assessments involve either counting of positive stained cells or demarcation of areas of damaged tissue. Often it is quite subjective as to where the border between what is damaged tissue and what is not, is drawn. We have investigated the use of Western blot analysis of injured spinal cords as a technique that could be used for objectively assessing changes in the levels of cellular markers induced by injury. Morphological analysis of tissue sections used for neuronal counts (see above) showed that at 24 hours and 4 days after injury immunoreactivity for NSE in particular (and for PGP9.5 to a lesser degree) was visible, but this was not associated with intact neuronal cell bodies, however, at 4 and 10 weeks after injury staining was only associated within intact cells. The Western blot analysis will detect all specific immunoreactive protein in the tissue sample regardless of whether it is associated with intact or disrupted cells. The western blot analysis showing progressive decrease in levels of cellular markers (CNPase, NSE and PGP9.5) up to 4 weeks is likely to reflect the clearance of non-cell associated markers. There were no further changes in the levels of any of the markers between 4 and 10 weeks which is consistent with our findings that there are no changes in either grey matter neurons or white matter axons after 4 weeks. The results show that using markers such as GFAP that are known to be upregulated following trauma may not provide a useful assessment of the size of injury (Fig. 8). Thus western blotting analysis seems to be best applicable for the estimate of spinal injury progression only for later stages after trauma.

### Impaired vascular supply following spinal cord injury

**Figure 10:**
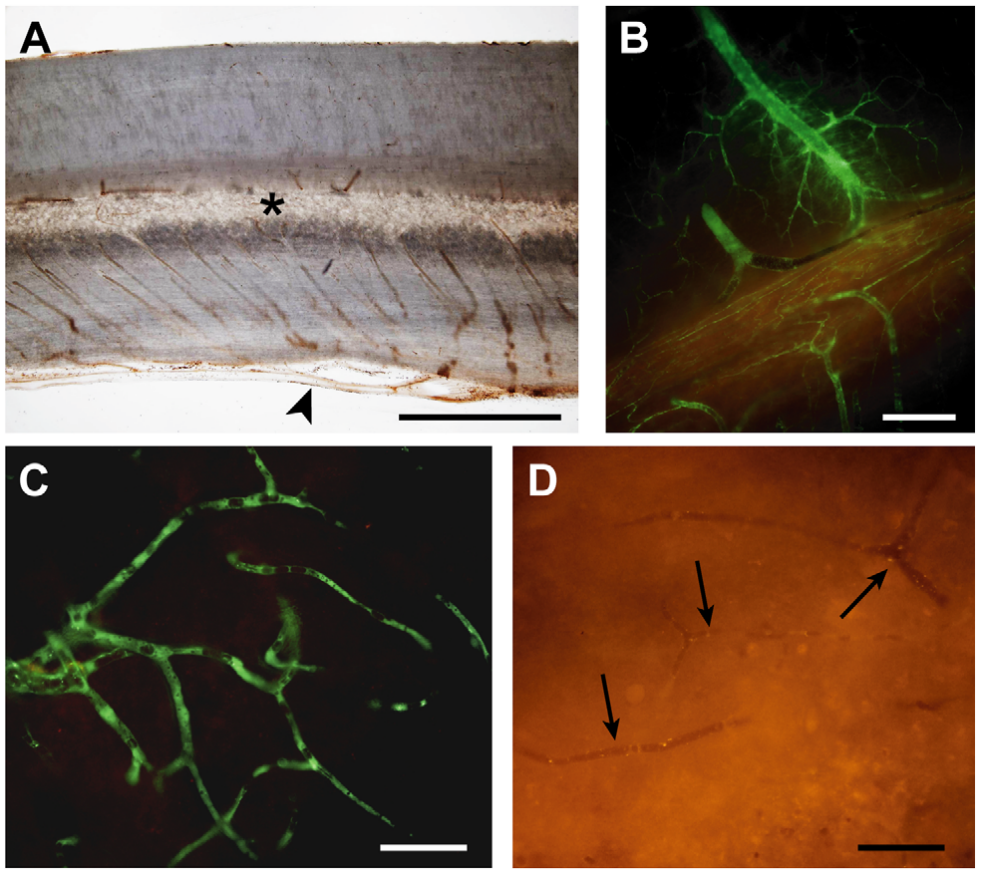
Vascular supply after spinal cord injury. **A**) Sagittal section (50 µm) of rat thoracic spinal cord showing the gross vascular anatomy. Note that parallel sulcal arteries, at every 50 to 100 µm along the cord, extending from the anterior spinal artery (arrowhead) into the central grey matter (*). This suggests a segmented vascular supply of the central grey matter. **B–D**) Sagittal sections (50 µm) of rat thoracic spinal cord 15 min after i.v. injection of a 70 kDa green fluorescent dextran into a rat 4 days after spinal injury. **B**) In this section from just outside the injury site, vessels are filled with the dextran indicating that blood flow is maintained in these vessels. **C**) Higher magnification shows that the dextran has reached small capillaries just outside the injury zone. This is in contrast to the injury site where capillaries are devoid of the dextran indicating that flow through these vessels is impeded (arrows in **D**). The highly segmental arrangement of the blood supply to the central grey matter (**A**) could account for the limited rostral and caudal expansion of the lesion since the blood supply to regions outside the initial injury zone appears to be maintained (**B** & **C**). Scale bars are 1 mm in **A**, 250 µm in **B** and 50 µm in **C** & **D**.

Some previous investigations of experimental spinal cord injury have suggested that the chief initiator of secondary tissue damage is the ischaemia that follows damage to the spinal vascular supply [7], [43]. Damage to blood vessels at the time of the injury occurs mainly in the central grey matter (Fig. 1D), but reductions in spinal cord blood flow in both grey matter and adjacent white matter up to several segments away from the injury site have been reported [11], [44], [45]. The central grey matter of the adult rat spinal cord receives most of its blood supply from the longitudinal anterior spinal artery and some from the posterior spinal arteries [46]–[49]. Numerous parallel sulcal arteries rise up from the anterior spinal artery and pass unbranched through the sulcal fissure (Fig. 10A) before branching profusely throughout the grey matter on one side of the spinal cord (rats [48], monkeys and cats [50]). In humans [8] and rats [48] adjacent sulcal arteries have been shown to alternately supply opposite sides of the spinal grey matter. Thus the blood supply to the central grey matter of the spinal cord appears to be a series of independent vascular units arranged in parallel along the length of the spinal cord. The deeper white matter adjacent to the central grey matter also receives its vascular supply mostly from blood vessels of sulcal origin, extending out from the central grey matter, whereas the outer white matter receives its supply mainly from blood vessels penetrating in from the pial surface. In the present experimental model, the impact to the dorsal aspect of the spinal cord compresses and ruptures a number of these small vascular units underneath and around the impactor tip, but probably does not affect vascular units located further away. Accordingly, grey matter further away from the impact site probably survives because its vascular supply remains intact and it thus experiences less ischaemia (compare Figs. 10B,C with 10D). This could account for the limited rostro-caudal spread of the lesion observed in this study.

### Behavioural analysis

The most widely used behavioural method of assessment has been the BBB score [15], [32] which suffers the drawbacks of being subjective and that the scaling is non-linear; this method does not clearly distinguish between behavioural improvements due to changes in supra-spinal and local spinal circuits. From 1 week after injury, the animals in the present study displayed some limited ability to walk. Their gait was grossly abnormal and the low BBB scores (Fig. 9A) indicate that this was probably dependent on local or reflex hindlimb function without supraspinal control. Subsequently the spinal injured rats gradually improved in their BBB scores up to 5 weeks after injury when scores reached a plateau of around 9 indicating plantar placement of the paw with weight support in stance only, or occasional, frequent, or consistent weight-supported stepping and no plantar stepping [15]. Scores of 8 or 9 indicate improvement at the spinal level, but not at the supra-spinal level. The majority of previous studies also report a plateau in scores between 3-6 weeks following spinal cord contusion injury [38], [51], [52]. A plateau in performance was also observed in the ledged beam test (Fig. 9B) and the random rung ladder test (Fig. 9C), which requires supraspinal control of hindlimb movements in order for the animal to avoid making slips. Spinal injured rats continued to make hindlimb errors over the entire testing period. Digital footprint analysis conducted at 4 and 10 weeks after injury corroborated a lack of improvement from 4 to 10 weeks in any measured hindlimb parameters Behavioural findings were therefore consistent with the data on lesion size.

The gradual improvement in BBB scores up to 5 weeks may be explained by recovery from the initial functional inactivity which typically follows severe spinal injury, or by establishment of local networks within the spinal cord or a combination of these. Establishment of local networks via propriospinal circuitry has been proposed as an explanation for the ability of spinal injured animals to produce walking movements when there is no evidence of projections from the brain past the lesion site [53], [54]. In quadrupeds, central pattern generators within the spinal cord are stimulated by stretch receptors in the muscles and cutaneous receptors in the paws of the hindlimbs, which automatically ‘walk’ in response to forward movements initiated by voluntary movement of the forelimbs [55]–[58]. These spinal controlled involuntary walking movements do not require cortico- or rubro-spinal tracts, or new local networks and can be generated in animals with complete spinal cord transection [53].

Despite the plateau in scores in all functional tests, we observed a general improvement in the appearance of the animals; they appeared more stable and less hesitant to move. Although digital footprint analysis showed no changes in hindlimb parameters between 4 and 10 weeks after injury we did find significant, presumably compensatory, alterations in the forelimbs of the rat in response to injury. Such alterations included narrowed step angle, increased stance width and reduced swing duration such that the rat kept its forelimbs further apart and on the ground for longer. Forelimb movements are not usually measured in thoracic injury studies except when determining co-ordination. Compensatory forelimb movements may also explain the improvement in BBB scores from weeks 1 to 5.

We also made an assessment of the response to nociceptive stimuli at different times after injury using the Von Frey hair test and reaction time in response to a plantar heat stimulus (unpublished). In the Von Frey test there were no significant differences between control and injured animals at either 4 or 10 weeks after injury. In the heat stimulus test, the reaction time at 2, 3 and 6–8 weeks post injury was 30 to 60% longer than controls. However, these differences were not statistically significant and as these tests do not distinguish between a delay in sensory or motor pathways, it is not clear if they would necessarily be a reflection of an abnormal sensory response.

### Conclusion

The results show that for the size of injury studied (which would be classified as “severe” when compared to other spinal cord contusion studies) the loss of grey matter is over by 24 hours after injury, but loss of white matter continues up to 1 week after injury. The rapid loss of grey matter neurons means that any therapeutic interventions aimed at limiting secondary damage to grey matter would need to be administered soon after an injury has occurred to be effective. Given that there is often a delay of several hours between when an injury occurs and when a patient arrives at a medical facility, the prospects for limiting secondary grey matter damage are not good unless these treatments could be commenced early by attending paramedics. In contrast, the slower progression of white matter damage suggests it is a more amenable target for therapeutic intervention in the first few days after injury.

## References

[1] Bramlett HM, Dietrich WD (2007). Progressive damage after brain and spinal cord injury: pathomechanisms and treatment strategies.. Prog Brain Res.

[2] Greve MW, Zink BJ (2009). Pathophysiology of traumatic brain injury.. Mt Sinai J Med.

[3] Park E, Bell JD, Siddiq IP, Baker AJ (2009). An analysis of regional microvascular loss and recovery following two grades of fluid percussion trauma: a role for hypoxia-inducible factors in traumatic brain injury.. J Cereb Blood Flow Metab.

[4] Rowland JW, Hawryluk GW, Kwon B, Fehlings MG (2008). Current status of acute spinal cord injury pathophysiology and emerging therapies: promise on the horizon.. Neurosurg Focus.

[5] Hawryluk GW, Rowland J, Kwon BK, Fehlings MG (2008). Protection and repair of the injured spinal cord: a review of completed, ongoing, and planned clinical trials for acute spinal cord injury.. Neurosurg Focus.

[6] Onose G, Anghelescu A, Muresanu DF, Padure L, Haras MA (2009). A review of published reports on neuroprotection in spinal cord injury.. Spinal Cord.

[7] Amar AP, Levy ML (1999). Pathogenesis and pharmacological strategies for mitigating secondary damage in acute spinal cord injury.. Neurosurg.

[8] Tator CH, Koyanagi I (1997). Vascular mechanisms in the pathophysiology of human spinal cord injury.. J Neurosurg.

[9] Yeo JD (1984). The use of hyperbaric oxygen to modify the effects of recent contusion injury to the spinal cord.. CNS Trauma: J Am Paralysis Assoc.

[10] Young W (1993). Secondary injury mechanisms in acute spinal cord injury.. J Emerg Med.

[11] Tator CH, Fehlings MG (1991). Review of the secondary injury theory of acute spinal cord trauma with emphasis on vascular mechanisms.. J Neurosurg.

[12] Bilgen M (2005). A new device for experimental modeling of central nervous system injuries.. Neurorehab Neural Repair.

[13] Narayana PA, Grill RJ, Chacko T, Vang R (2004). Endogenous recovery of injured spinal cord: longitudinal in vivo magnetic resonance imaging.. J Neurosci Res.

[14] Bradford MM (1976). A rapid and sensitive method for the quan- titation of microgram quantities of protein utilizing the principle of protein-dye binding.. Anal Biochem.

[15] Basso DM, Beattie MS, Bresnahan JC (1995). A sensitive and reliable locomotor rating scale for open field testing in rats.. J Neurotrauma.

[16] Schallert T, Bland ST, Fleming SM, Krieglstein J, Klumpp S (2000). Functional recovery after brain injury: role of neurotrophic factors and behavior-driven structural events.. Pharmacology of Cerebral Ischemia.

[17] Kakoulas BA (2004). Neuropathology: the foundation for new treatments in spinal cord injury.. Spinal Cord.

[18] Busch SA, Horn KP, Silver DJ, Silver J (2009). Overcoming macrophage-mediated axonal dieback following CNS injury.. J Neurosci.

[19] Hollis ER, Lu P, Blesch A, Tuszynski MH (2009). IGF-I gene delivery promotes corticospinal neuronal survival but not regeneration after adult CNS injury.. Exp Neurol.

[20] Oschipok LW, Teh J, McPhail LT, Tetzlaff W (2008). Expression of Semaphorin3C in axotomized rodent facial and rubrospinal neurons.. Neurosci Lett.

[21] Wannier-Morino P, Schmidlin E, Freund P, Belhaj-Saif A, Bloch J (2008). Fate of rubrospinal neurons after unilateral section of the cervical spinal cord in adult macaque monkeys: effects of an antibody treatment neutralizing Nogo-A.. Brain Res.

[22] Chen Y, Constantini S, Trembovler V, Weinstock M, Shohami E (1996). An experimental model of closed head injury in mice: patho- physiology, histopathology, and cognitive deficits.. J Neurotrauma.

[23] Lonjon N, Kouyoumdjian P, Prieto M, Bauchet L, Haton H (2010). Early functional outcomes and histological analysis after spinal cord compression injury in rats.. J Neurosurg Spine.

[24] Habgood MD, Bye N, Dziegielewska KM, Ek CJ, Lane MA (2007). Changes in blood-brain barrier permeability to large and small molecules following traumatic brain injury in mice.. Eur J Neurosci.

[25] Allen AR (1911). Surgery of experimental lesion of spinal cord equivalent to crush injury of fracture dislocation of spinal column. A preliminary report.. J Am Med Assoc.

[26] Gruner JA (1992). A monitored contusion model of spinal cord injury in the rat.. J Neurotrauma.

[27] Young W (2002). Spinal cord contusion models.. Prog Brain Res.

[28] Wrathall JR, Pettegrew RK, Harvey F (1985). Spinal cord contusion in the rat: production of graded, reproducible, injury groups.. Exp Neurol.

[29] Noyes DH (1987). Correlation between parameters of spinal cord impact and resultant injury.. Exp Neurol.

[30] Stokes BT, Noyes DH, Behrmann DL (1992). An electromechanical spinal injury technique with dynamic sensitivity.. J Neurotrauma.

[31] Scheff SW, Rabchevsky AG, Fugaccia I, Main JA, Lumpp JE (2003). Experimental modeling of spinal cord injury: characterization of a force-defined injury device.. J Neurotrauma.

[32] Basso DM, Beattie MS, Bresnahan JC (1996). Graded histological and locomotor outcomes after spinal cord contusion using the NYU weight-drop device versus transection.. Exp Neurol.

[33] Ghasemlou N, Kerr BJ, David S (2005). Tissue displacement and impact force are important contributors to outcome after spinal cord contusion injury.. Exp Neurol.

[34] Kim J, Tu TW, Bayly P, Song SK (2009). Impact speed does not determine severity of spinal cord injury in mice with fixed impact displacement.. J Neurotrauma.

[35] Constantini SC, Young W (1994). The effects of methylprednisolone and the ganglioside GM1 on acute spinal cord injury in rats.. J Neurosurg.

[36] Bresnehan JC, Beattie MS, Todd FD, Noyes DH (1987). A behavioral and anatomical analysis of spinal cord injury produced by a feedback-controlled impaction device.. Exp Neurol.

[37] Pearse DD, Lo TP, Cho KS, Lynch MP, Garg MS (2005). Histopathological and behavioral characterization of a novel cervical spinal cord displacement contusion injury in the rat.. J Neurotrauma.

[38] Pinzon A, Marcillo A, Quintana A, Stamler S, Bunge MB (2008). A re-assessment of minocycline as a neuroprotective agent in a rat spinal cord contusion model.. Brain Res.

[39] Noble LJ, Wrathall JR (1989). Correlative analyses of lesion development and functional status after graded spinal cord contusive injuries in the rat.. Exp Neurol.

[40] Andrade MS, Hanania FR, Daci K, Leme RJ, Chadi G (2008). Contuse lesion of the rat spinal cord of moderate intensity leads to a higher time-dependent secondary neurodegeneration than severe one. An open-window for experimental neuroprotective interventions.. Tissue Cell.

[41] Balentine JD, Paris U (1978). Pathology of experimental spinal cord trauma. I. The necrotic lesion as a function of vascular injury.. Lab Invest.

[42] Casella GT, Bunge MB, Wood PM (2006). Endothelial cell loss is not a major cause of neuronal and glial cell death following contusion injury of the spinal cord.. Exp Neurol.

[43] Tator CH (1992). Hemodynamic issues and vascular factors in acute experimental spinal cord injury.. J Neurotrauma.

[44] Rivlin AS, Tator CH (1978). Regional spinal cord blood flow in rats after severe cord trauma.. J Neurosurg.

[45] Guha A, Tator CH, Rochon J (1989). Spinal cord blood flow and systemic blood pressure after experimental spinal cord injury in rats.. Stroke.

[46] Woollam DH, Millen JW (1955). The arterial supply of the spinal cord and its significance.. J Neurol Neurosurg Psychiatr.

[47] Wallace MC, Tator CH, Frazee P (1986). Relationship between posttraumatic ischemia and hemorrhage in the injured rat spinal cord as shown by colloidal carbon angiography.. Neurosurg.

[48] Koyanagi I, Tator CH, Lea PJ (1993). Three-dimensional analysis of the vascular system in the rat spinal cord with scanning electron microscopy of vascular corrosion casts. Part 1: Normal spinal cord.. Neurosurg.

[49] Koyanagi I, Tator CH, Theriault E (1993). Silicone rubber microangiography of acute spinal cord injury in the rat.. Neurosurg.

[50] Lange W, Leonhardt H (1978). Paired vessels in the spinal cord of Rhesus monkey and cat.. Anat Embryol.

[51] Koopmans GC, Deumens R, Buss A, Geoghegan L, Myint A (2009). Acute rolipram/thalidomide treatment improves tissue sparing and locomotion after experimental spinal cord injury.. Exp Neurol.

[52] Hamers FP, Lankhorst AJ, van Laar TJ, Veldhuis WB, Gispen WH (2001). Automated quantitative gait analysis during overground locomotion in the rat: its application to spinal cord contusion and transection injuries.. J Neurotrauma.

[53] Bareyre FM, Kerschensteiner M, Raineteau O, Mettenleiter TC, Weinmann O (2004). The injured spinal cord spontaneously forms a new intraspinal circuit in adult rats.. Nat Neurosci.

[54] Courtine G, Song B, Roy RR, Zhong H, Herrmann JE (2008). Recovery of supraspinal control of stepping via indirect propriospinal relay connections after spinal cord injury.. Nat Med.

[55] Grillner S, Wallé P (1985). Central pattern generators for locomotion, with special reference to vertebrates.. Annu Rev Neurosci.

[56] Edgerton VR, Roy RR, Hodgson JA, Prober RJ, de Guzman CP (1992). Potential of adult mammalian lumbosacral spinal cord to execute and acquire improved locomotion in the absence of supraspinal input.. J Neurotrauma.

[57] Rossignol S, Barbeau H (1993). Pharmacology of locomotion: an account of studies in spinal cats and spinal cord injured subjects.. J Am Paraplegia Soc.

[58] Muir GD, Steeves JD (1995). Phasic cutaneous input facilitates locomotor recovery after incomplete spinal injury in the chick.. J Neurophysiol.

